# Recovery of 52 bacterial genomes from the fecal microbiome of the domestic cat (*Felis catus*) using Hi-C proximity ligation and shotgun metagenomics

**DOI:** 10.1128/MRA.00601-23

**Published:** 2023-09-11

**Authors:** Connie A. Rojas, Jennifer Gardy, Jonathan A. Eisen, Holly H. Ganz

**Affiliations:** 1 AnimalBiome, Oakland, California, USA; 2 Bill & Melinda Gates Foundation, Seattle, Washington, USA; 3 Evolution and Ecology, University of California, Davis, California, USA; University of Maryland School of Medicine, Baltimore, Maryland, USA

**Keywords:** gut microbiome, fecal microbiome, metagenome-assembled genomes (MAGs), domestic cats, gut bacteria, assembly, shotgun metagenomics, Hi-C, antimicrobial-resistance

## Abstract

We used Hi-C proximity ligation with shotgun sequencing to retrieve metagenome-assembled genomes (MAGs) from the fecal microbiomes of two domestic cats (*Felis catus*). The genomes were assessed for completeness and contamination, classified taxonomically, and annotated for putative antimicrobial resistance (AMR) genes.

## ANNOUNCEMENT

Domestic cats (*Felis catus*) are obligate carnivores (1) that rely on their gut microbiome for digestion (2–4). Prior studies have mapped the composition of the fecal microbiome in cats (5–8), but no complete bacterial genomes have been reconstructed using metagenomic sequencing. One promising approach is Hi-C technology, which cross-links, digests, and ligates DNA (9) to improve genome assembly. Here we used metagenomic sequencing with Hi-C proximity ligation to reconstruct genomes (MAGs) from two cats and assess the usefulness of this approach for detecting bacteria that may be novel.

We collected fresh fecal material from two domestic shorthairs (Danny and Lil Bub). Danny was a healthy, 9-yr-old neutered male who lived indoors. Lil Bub was an indoor, 4-yr-old spayed female celebrity cat with osteopetrosis. The UC Davis IACUC determined that formal approval was not necessary because fecal material is a waste product.

DNA was isolated from fecal samples using the MoBio PowerFecal DNA Extraction Kit. A Hi-C library was created with the Phase Genomics (Seattle, WA, USA) ProxiMeta Hi-C v4.0 Kit (10). A metagenomic shotgun library was also prepared with ProxiMeta reagents. Sequencing was performed on an Illumina NovaSeq generating PE reads (150 bp) for both Hi-C and shotgun libraries. Sequencing files were uploaded to the Phase Genomics portal for quality filtering with fastp (v0.23.2) (11) and assembly with MEGAHIT (v1.2.9) (12). Assembly quality was assessed with QUAST (v5.0) (13). For these tools and subsequent tools, default parameters were used unless otherwise specified. Hi-C reads were aligned to the assembly using BWA-MEM (v0.7.17) (14). Metagenome deconvolution was performed with ProxiMeta (15, 16), and the resulting MAGs were assessed for quality using CheckM (v1.2.0) (17) and assigned taxonomy with the Genome Taxonomy Database release 202 (gtdbtk v.1.5.0) (18). Antimicrobial resistance (AMR) genes were annotated with the NCBI AMRFinderPlus (v3.10.5) (19).

We recovered a total of 52 MAGs with >90% completeness and <5% contamination and nine MAGs with >80% completeness and <5% contamination. The most common bacterial families were *Lachnospiraceae*, *Oscillospiraceae*, *Bacteroidaceae*, and *Erysipelotrichaceae*. Seventeen MAGs were assigned a species-level classification, and among them were *Alistipes putredinis*, *Bacteroides finegoldii*, *Campylobacter helveticus*, *Collinsella tanakaei*, *Parabacteroides merdae*, *Prevotella copri,* and *Ruminococcus gnavus*.

A total of 31 unique antimicrobial-resistance genes (AMRs) were detected in contigs and were predicted to encode resistance to tetracycline, erythromycin, kanamycin, and beta-lactam antibiotics. Multidrug efflux transporters, beta-lactamases, and methyltransferases were among the putative proteins.

Our data is limited to two individual cats, but we add to the growing catalog of studies using Hi-C with shotgun sequencing to retrieve quality genomes from mammals, including dogs (20), sheep (21), cows (16), and humans (22).

**TABLE 1:** Overview of data files/data sets

Data description	File types (file extension)	Links
Hi-C Illumina Sequences (PE 150 bp) from Danny (973,507 reads)	Fastq file (.fastq.gz)	*NCBI Sequence Read Archive* https://identifiers.org/ncbi/insdc.sra:SRX18058393
Hi-C Illumina Sequences (PE 150 bp) from Lil Bub (14,782,886 reads)	Fastq file (.fastq.gz)	*NCBI Sequence Read Archiv*e https://identifiers.org/ncbi/insdc.sra:SRX18058392
Assembled Contigs from Illumina Shotgun Sequences (Danny) (62,536 contigs)	Fasta file (.fa)	*NCBI GenBank* https://identifiers.org/nucleotide:JAPFCN000000000.1
Assembled Contigs from Illumina Shotgun Sequences (Lil Bub) (82,672 contigs)	Fasta file (.fa)	*NCBI GenBank* https://identifiers.org/nucleotide:JAPFCM000000000.1
Statistics on the Assembled Contigs using QUAST	Word document (.docx)	*Figshare* https://doi.org/10.6084/m9.figshare.23827035
MAG fasta files	Fasta file (.fa)	*NCBI BioProject* https://identifiers.org/ncbi/bioproject:PRJNA893230
List of MAGs, their taxonomy, and basic statistics	Excel spreadsheet (.xlsx)	*Figshare* https://doi.org/10.6084/m9.figshare.21555165
More detailed study methods	Word document (.docx)	*Figshare* https://doi.org/10.6084/m9.figshare.21440763.v6

## Data Availability

All genomic sequence data is under NCBI BioProject PRJNA893230. Raw Hi-C Illumina sequences are in the Sequence Read Archive (accessions SRR22078165 and SRR22078166), unannotated assemblies are in NCBI GenBank (accessions JAPFCN010000000 and JAPFCM010000000), and MAGs are also in NCBI GenBank. Please refer to Table 1 for links to the data.
